# Editorial: Population genomic architecture: Conserved polymorphic sequences (CPSs), not linkage disequilibrium

**DOI:** 10.3389/fgene.2023.1140350

**Published:** 2023-01-26

**Authors:** Chester A. Alper, Roger L. Dawkins, Jerzy K. Kulski, Charles E. Larsen, Sally S. Lloyd

**Affiliations:** ^1^ Program in Cellular and Molecular Medicine, Boston Children’s Hospital, Boston, MA, United States; ^2^ Department of Pediatrics, Harvard Medical School, Boston, MA, United States; ^3^ CY O’Connor ERADE Village Foundation, North Dandalup, WA, Australia; ^4^ Department of Molecular Life Sciences, Division of Basic Medical Science and Molecular Medicine, Tokai University School of Medicine, Isehara, Japan

**Keywords:** haplotype, genomic architecture, polymorphism, major histocompatibility complex, linkage disequilibrium, recombination, pedigree

The human extended major histocompatibility complex (MHC) region, ∼7.7 megabases (Mb) in the middle of the short arm of chromosome 6, is one of the most gene-dense and polymorphic regions of the human genome (Horton et al., 2004; Shiina et al., 2009; Kulski et al., 2022). MHC haplotype characterization originated largely from genetic studies in the 1960s–1990s by Ceppellini et al. (1967) and the research groups of Chester Alper (Alper et al., 1983; Awdeh et al., 1983; Yunis et al., 2003) and Roger Dawkins (Degli-Esposti et al., 1992; Degli-Esposti et al., 1995; Dawkins et al., 1999).

In this Research Topic, we introduced the term “conserved polymorphic sequences” (CPSs) to encompass both Mb-length haplotypes, labeled currently either as conserved extended haplotypes or ancestral haplotypes, and their haplotypic fragments (“fixed” or “frozen” blocks). The relevance of CPSs includes: 1) an improved understanding of MHC genomic architecture--laying the foundation for other regions; 2) their use in testing genetic models for both meiotic recombination and trait association; and 3) providing insight into population genetics and evolution. Linkage disequilibrium (LD) and other expectation-maximization (EM) analyses of unrelated genotypes fail to detect most of the larger CPS variants but often detect their small (5–200 kb) blocks (Walsh et al., 2003; Alper et al., 2006). The technical explanation(s) for these differences remain unresolved. The Research Topic includes original work, a minireview, a commentary, and a mechanistic model for CPSs at high population frequency. The CPS mechanistic model may apply to any genomic region or vertebrate species (Dawkins and Lloyd).

Alper reviewed CPS conceptual development. The identification of high population frequency Mb-length MHC haplotypes resulted from observed shorter (140 kb) MHC-encoded complement gene haplotypes (“complotypes”; Alper et al., 1983) in strong LD with HLA class I and class II haplotypic variants. Later publications demonstrated both the intervening genetic “fixity” (essentially fixed DNA sequence) among each of those long-range population haplotypes and their extension from the telomeric end of the HLA class I region to the centromeric end of HLA class II (e.g., Dawkins et al., 1999; Smith et al., 2006; Lam et al., 2015). Alper concluded that the CPS concept offers new genomic insights (Larsen et al., 2014; Vadva et al., 2019) and genetic/epigenetic models for complex genetic traits (Alper et al., 2019).

Okano et al. described MHC CPSs in the domestic cat and highlighted the differences between human and cat MHC haplotype organization, consistent with prior work showing divergent vertebrate MHC haplotype structures (Kulski et al., 2002; Shiina et al., 2017). Using new genotyping methods and separate 3-generation cat pedigrees, the authors identified 14 unrelated long-range (within the 3 Mb domestic cat MHC reference sequence) FLA-I/FLA-DRB allelic variant founder haplotypes. Eight unique variants were identified, only three of which were singletons. One CPS variant consisted of three founder haplotypes, and the remaining four existed twice.

Specific CPS variant frequencies differ considerably among human ethnic populations, and genetic studies of a wider range of populations are needed (Sirugo et al., 2019). Two papers expanded CPS studies to under-studied human populations. Tay et al., using segregation analysis to phase long-range HLA haplotypes in families with at least one type 1 diabetes (T1D) proband from the United Arab Emirates (UAE), reported the classical association of HLA-DR4 and HLA-DR3 haplotypes in UAE citizens affected by T1D. UAE T1D patient haplotypes with those HLA class II specificities were enriched in two Mb-length CPSs previously reported in northern India. This report preceded another pedigree-phased HLA haplotype analysis of 41 families (Alnaqbi et al., 2022) that identified three novel UAE population MHC CPSs.

Among twelve minority ethnic populations in China, Cun et al. reported on HLA class II haplotypes containing *HLA-DRB1* and five polymorphic Alu insertions (POALINs; a structural variant type of transposable element (TE) genetic marker) covering 850 kb. These HLA class II TE markers of diversity and historic crossing over showed strong association among these populations with known language family, migration, and sociality characteristics. The results were compared with those obtained from dominant populations from Japan and Australia (Kulski et al., 2010) and the Yunnan province of China (Shi et al., 2014).

Mb-length CPSs constitute nearly half of European Caucasian MHC haplotypes (Szilágyi et al., 2010). However, to identify the diversity of all haplotypes, relatively dense genetic markers are needed to detect genomic alterations (e.g., meiotic recombination, insertion-deletion polymorphisms). As part of an investigation into putative associations and functions of TEs in generating human (Kulski et al., 1997; Kulski et al., 1999; Kulski et al., 2000) and non-human primate (Kulski et al., 2004) MHC haplotypes, two reports by Kulski, Suzuki and Shiina used a set of 95 extended MHC haplotype sequences from a publicly available database (Norman et al., 2017) to study meiotic recombination resulting in haplotype segmental shuffling or crossovers in the MHC class I and II regions. Both studies demonstrated that 1) SNP-density crossovers are associated with putative ancestral recombination sites widely spread across the odortype receptor and MHC class I and class II regions; 2) MHC homozygous cell line genomic sequences are useful for analyzing haplotype blocks, ancestral haplotypic landscapes and markers, CPSs, and SNP-density crossover junctions without the need for probabilistic statistical imputation; and 3) TEs are useful genetic markers of recent recombination events and for elucidating population phylogenetics and genetic interrelationships (Kulski et al., 2011; 2019; Wang et al., 2017; Abeid et al., 2019). There was substantial haplotype shuffling between different polymorphic blocks suggesting the presence of numerous putative ancestral recombination sites between specific class II genes. TEs, in addition to being a useful class of haplotypic markers, may have had a critical impact on CPS diversity between individuals and population groups.

Finally, Radman offered a hypothesis, which was further contextualized in a commentary by Dawkins and Lloyd, for the existence of long-range CPSs: as a consequence of resistance to meiotic recombination both in regions with highly polymorphic sequences and in regions dense with gene families requiring maintenance of genomic integrity. Significantly, the MHC class III region (between HLA class I and class II) is both the most gene-dense sub-region of the extended MHC (Horton et al., 2004) and, along with a portion of the telomeric end of class II, the most difficult MHC region to align sequencing assemblies due to high levels of indel polymorphism (Horton et al., 2008; Chin et al., 2020). Radman’s mechanistic model and this Research Topic are pleas for additional work to understand better the enigmatic relationships between CPS haplotypes, recombination mechanisms, and genetic fixity with genomic structural integrity and diversity within and between different populations and species. A database (http://cyo.edu.au/CPS_Database) is available for researchers wishing to submit CPSs of 400 nucleotides or longer shown to exist as at least three variants within a specific genome at a well-defined genomic location.
